# Two Remarkable Cases of Intermittent Aphonia

**Published:** 1829-10-01

**Authors:** 


### XXVI.

TWO REMARKABLE CASES OF INTERMITTENT APHONIA. By Messrs. RENNES and OLLIVIER.

*Case* 1. The case is related by M. Rennes, Professor of Medicine at Strasbourg. The patient was a married lady, aged 46 years, who resided in a healthy part of the country, and had never experienced any material illness. This female was attacked annually, from the age of 33 years, with aphonia. These attacks were abrupt, and came on at noon, unaccompanied, at first, with any local or general uneasiness. The loss of voice was sudden and complete, and lasted till midnight of the same day, when speech returned in the same rapid manner in which it was lost. This occurred every day for about three weeks, and then ceased. Three of these accessions, each of a fortnight or three weeks' duration, took place in the course of the first year, 1812, and the same course obtained for a few years afterwards. In 1819, the periodicity became more regular. Each year, in the month of February, the approach of the malady was announced, in the morning, by wandering pains, shiverings, and horripilations. Exactly at mid-day the voice became extinct, without any affection of the pulse, any sense of constriction about the throat—but only a sense of tightness and malaise in the epigastric region. She would go to bed at nine o'clock, and awake quite well—to go through the same process the succeeding day. Sometimes, however, there were accompanying febrile symptoms, head-ache, &c. but these were rare. She had no perspiration. Nevertheless these attacks diminished her strength, and caused considerable loss of flesh. She had been bled—had taken sulphate of quinine, and various other medicines, without the least effect. Of late years, the duration of these attacks has varied from three to seven months every year. The attack over, she regains her embonpoint and strength, and appears as if she never had been ailing.

*Case* 2. This is entitled by M. Ollivier an intermittent aphonia, of 30 years standing, always cured (for a time) by venesection. Maria Louisa Giron, midwife at the Hôtel Dieu of Angers, aged 44 years, at the time she came under observation, 1818, of nervous and sanguineous temperament, had menstruated regularly from the age of 15 till that of 18, when she had a profuse menorrhagia, occasioned by some violent mental emotions. About this period she became affected for the first time, and without any evident cause, with complete aphonia, which lasted several days. From this time the complaint came on at irregular intervals—sometimes several times in the month—sometimes with intervals of many months. She had once an immunity of a whole year. There was no apparent disturbance of the constitution at the periods of attack. They commenced with a sense of irritation in the throat, which gradually increased till the voice was totally lost, when the irritation always subsided. During the period of the attack, the patient felt a sense of oppression about the region of the heart, and of heat ascending in currents to the head. In all other respects she was well, and went about her avocations as usual. From the age of 15 years up to that of 44 years, the attacks of aphonia have always presented the same phenomena; but latterly that menstruation has ceased, the intervals between the accessions are longer. Various remedies, chiefly of the antispasmodic and tonic kind, had been employed, without making the slightest impression on the malady. At length blood-letting was tried—and scarcely had an ounce of blood flowed from a vein when the voice became restored. The same remedy has invariably produced the same effect, in every subsequent attack of the aphonia. Leeches gave the same relief, by the time that an ounce or two of blood were abstracted. M. Ollivier himself was witness many times of this extraordinary efficacy of bleeding. The patient felt as if a load were removed from about her heart whenever the blood flowed, and then the voice was restored.

The above cases are very curious—but especially the latter, in a therapeutical point of view. The remedy is worthy of trial in cases of aphonia. In respect to the first case, it is said by M. Rennes that the patient inhabited a place where the "air was good, and generally salutary"—but this place we find to be, "sa maison de campagne situé dans le Perigord, *au milieu des bois*." Now a house pitched in the midst of woods, must be one where there was very probably a plentiful supply of malaria, or vegeto-animal emanations; and we cannot but consider the case, after all, as one belonging to the ancient and extensive family of the Agues. *Archives.*

---

XXVII.

